# Revolutionizing tuberculosis treatment: Breakthroughs, challenges, and hope on the horizon

**DOI:** 10.1016/j.apsb.2025.01.023

**Published:** 2025-01-31

**Authors:** Martin Kufa, Vladimir Finger, Ondrej Kovar, Ondrej Soukup, Carilyn Torruellas, Jaroslav Roh, Jan Korabecny

**Affiliations:** a Faculty of Pharmacy in Hradec Kralové, Charles University, Hradec Kralove 50003, Czech Republic; b Biomedical Research Center, University Hospital Hradec Kralove, Hradec Kralove 50003, Czech Republic; c U.S. Army CCDC Chemical Biological Center, MD 21010-5424, USA

**Keywords:** Antibiotic resistance, Clinical trials, Drug discovery, *Mycobacterium tuberculosis*, Tuberculosis

## Abstract

Tuberculosis (TB), an infectious disease caused by the bacterium *Mycobacterium tuberculosis* (*Mtb*), was responsible for the deaths of approximately 1.3 million people in 2022. In addition, 7.5 million new cases of TB have been reported. Present-day treatments require a daily dosing of a multiple-drug regimen for a minimum of six-month, but poor adherence and other factors often lead to treatment failure. Consequently, drug-resistant TB strains have become a growing concern, leading to more complex and expensive treatments. Promising drugs such as bedaquiline, delamanid, and pretomanid have been recently released, and 19 drug candidates are currently at different phases of clinical trials, addressing the problem of drug-resistant TB. Notwithstanding recent advances, the development of effective and safe drugs with novel mechanisms of action remains a challenge due to the unique nature of *Mtb*. Despite the persistent need for new treatments, TB research remains underfunded, highlighting the importance of collaborations between academia and the private sector in the advancement of anti-TB drug development. This review provides a perspective on the dynamic landscape of anti-TB drug discovery in recent years, offering hope for a more effective approach to combat this persistent global health threat.

## Graphical abstract

This review provides a perspective on the dynamic landscape of anti-tubercular drug discovery, offering hope for a more effective approach to combat this persistent global health threat.

**Figure:**
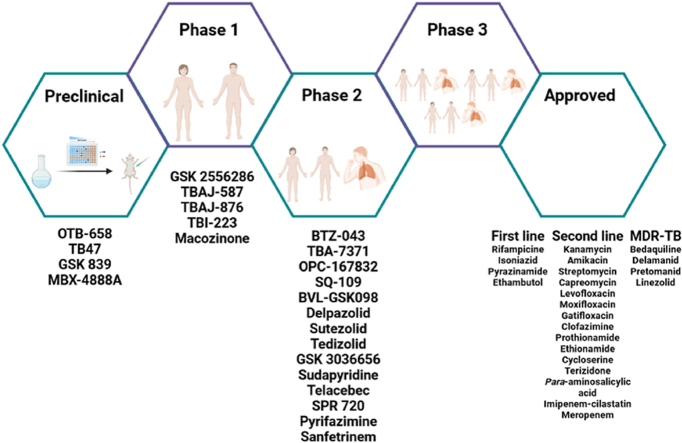

## 1 Introduction

Tuberculosis (TB) is a transmissible infectious disease caused by the intracellular bacteria *Mycobacterium tuberculosis* (*Mtb*)^1^. TB stands as one of the principal causes of mortality in low and middle-income countries^2^. TB is primarily a pulmonary infection that is able to disseminate to other parts of the body. Although it is transmitted *via* airborne particles released by active TB patients, *Mtb* can also survive in the host for a long time in an asymptomatic form known as latent TB infection (LTBI). Those with LTBI are not infectious, but the LTBI can transform into active TB when the immunity system is compromised by other diseases such as human immunodeficiency virus (HIV)^3^. In 2022, 7.5 million newly diagnosed TB infection and 1.3 million deaths were recorded. Remarkably, 410,000 patients developed drug-resistant TB (DR-TB)^1^, while LTBI prevailed in 25% of the World's population^3^.

If inadequately treated, the active form of TB is often fatal, reaching a mortality rate up to 50%^4^. The standard treatment regimens for drug-susceptible TB (DS-TB) is driven by module 4 of the World's Health Organization (WHO) guidelines released in 2022, recommending daily dosing of the first-line drug for six months. During the first two months, isoniazid (INH), rifampicin (RIF), ethambutol (EMB) and pyrazinamide (PZA) are administered, followed by four months of INH and RIF therapy. This drug combination is successful in 85% of cases^5^. The failure of DS-TB treatment is caused by many factors: poor patient adherence, alcohol consumption, smoking, and co-infections. On the other hand, patients under 65 years of age, and females favor the success of TB treatment^6^. *Mtb* can quickly develop resistance against anti-TB drugs. If not cured adequately, DS-TB strains can convert into DR-TB. WHO categorizes these strains into five groups based on their resistance patterns: INH resistant TB (IR-TB); RIF resistant TB (RR-TB); strains resistant to both INH and RIF are referred to as multidrug-resistant (MDR-TB); strains resistant to RIF and any fluoroquinolone (FQ), a broad-spectrum bactericidal antibiotics, are referred to as pre-extensively drug-resistant (pre-XDR-TB). The last category is extensively drug-resistant strains (XDR-TB) showing resistance to RIF, any FQ, plus at least one of the following drugs: bedaquline (BDQ) or linezolid (LNZ)^7^^,^^8^. These strains pose significant problem as they necessitate treatment with second-line drugs, which are associated with frequent and severe side effects, and higher costs^9^.

In 2016, WHO recommended two regimens for the treatment of DR-TB infections: a standardized 9 to 12-month regimen and an individualized regimen lasting ≥20 months. The standardized regimen (Table 1) is used for patients who have not been treated with any second-line drug previously but are anticipated to be infected with strains sensitive to FQ and second-line injectable agents. In cases where the drugs in the standardized regimen cannot be administered, WHO recommends an individualized regimen (Table 2, revised in 2019)^10^^,^^11^. The most recent addition to the arsenal of anti-TB drugs is pretomanid, introduced to the WHO guidelines in 2020. In 2022, pretomanid became part of the newly recommended 6-month regimen BPaLM (BDQ, pretomanid, LNZ, and moxifloxacin) for the treatment of MDR/RR-TB and pre-XDR-TB^5^.

**Table 1:** Anti-TB drugs used in standardized MDR-TB treatment regimens and their mechanisms of action (MOA). The MOA for each drug is shown once only.

Phase	Drug	MOA (in *Mtb*)
Intensive (4–6 months)	Gatifloxacin or moxifloxacin	DNA gyrase inhibition
	Kanamycin	Proteosynthesis inhibition
	Prothionamide (PTH)	Mycolic acid biosynthesis inhibition
	Clofazimine	Binding to DNA, inhibition of bacterial proliferation
	High-dose isoniazid	Mycolic acid biosynthesis inhibition
	Pyrazinamide	The MOA is unknown. A potential mechanism may involve the inhibition of CoA biosynthesis.
	Ethambutol	Arabinogalactan biosynthesis inhibition
Continuation for additional 5 months	Gatifloxacin or moxifloxacin	
	Clofazimine	
	Pyrazinamide	
	Ethambutol

**Table 2:** Anti-TB drugs recommended by WHO for individualized or long-term MDR-TB therapy with known or anticipated MOA. The MOA for each drug is shown once only.

Group (recommendation)	Drug	MOA (in *Mtb*)
Group A (all three agents are used in combination)	Levofloxacin or moxifloxacin	DNA gyrase inhibition
	Bedaquiline	Inhibition of ATP synthase
	Linezolid	Proteosynthesis inhibition
Group B (one or both agents are applied)	Clofazimine	
	Cycloserine or terizidone	Peptidoglycan biosynthesis inhibition
Group C (applied to complete the regimen and when drugs from Group A and B cannot be used)	Ethambutol	
	Delamanid	Mycolic acid biosynthesis inhibition
	Pyrazinamide	
	Imipenem-cilastatin or meropenem	Peptidoglycan biosynthesis inhibition
	Amikacin or streptomycin	Proteosynthesis inhibition
	Ethionamide (ETH) or PTH	Mycolic acid biosynthesis inhibition
	*Para*-aminosalicylic acid	Inhibition of folate metabolism

Even in the case of DS-TB, the treatment for TB is a lengthy process, contributing to the emergence of drug-resistant strains. TB drug-resistance poses an imminent threat to developing countries, particularly in the context of globalization. For these reasons, the search for more effective drugs and shorter treatments become a critical task, yet TB research remains underfunded. In 2022, approximately $1 billion was allocated to TB research, half the annual target set by the member states of the United Nations during the 2018 high-level meeting on the Fight to End TB^12^.

Despite the financial challenge, three new anti-TB agents: BDQ^13^, delamanid^14^ and pretomanid^15^ have been approved. Moreover, 19 small molecule drugs are currently under investigation in clinical trials, with another four candidates in preclinical testing with prospects to enter clinical trials (Table 3). These advancements hold great promise, although resistance to some of these drugs has emerged shortly after their introduction into clinical practice^16^. The continuous search for safe anti-TB molecules is crucial to curb the spread of DR-TB.

**Table 3:** Anti-TB agents in clinical trials. Chemical structures, their target and the National Clinical Trial (NCT) identifier numbers are provided. Trial status is disclosed directly to NCT number.

Name	Structure	Target	Phase in clinical trial^a^
BTZ-043		DprE1 (cell wall biosynthesis)	IIb/IIc NCT04044001 completed NCT03590600 completed NCT04874948 completed NCT05926466 recruiting NCT06114628 recruiting NCT05382312 recruiting NCT05807399 recruiting
Macozinone (PBTZ-169)		DprE1	Ib/IIa NCT04150224 completed with results NCT03423030 completed NCT03036163 completed with results NCT03776500 completed NCT03334734 terminated
TBA-7371		DprE1	IIa NCT03199339 completed NCT04176250 completed
OPC-167832		DprE1	IIb/IIc NCT03678688 completed with results NCT05221502 active, not recruiting NCT05971602 recruiting
SQ-109		MmpL3 (cell wall biosynthesis) MenA, MenG (energy metabolism)	IIb NCT01218217 completed NCT00866190 completed NCT01585636 completed NCT01358162 completed NCT01785186 completed with results NCT01874314 withdrawn
BVL-GSK098		Transcriptional regulator (activity boosting of ETH and PTH)	IIa NCT04654143 completed NCT05473195 recruiting
Delpazolid		50*S* ribosomal subunit (interfering with proteosynthesis)	IIb NCT04550832 completed NCT02836483 completed NCT01554995 completed NCT02538003 completed NCT02529241 terminated NCT04939779 completed NCT01842516 completed NCT02540460 completed NCT02882789 completed NCT03492996 completed
Sutezolid		50*S* ribosomal subunit	IIb/c NCT03199313 completed NCT03959566 completed NCT00990990 completed NCT01225640 completed NCT00871949 completed NCT05686356 recruiting NCT03237182 terminated NCT06192160 not yet recruiting NCT05971602 recruiting
TBI-223		50*S* ribosomal subunit	Ib NCT03758612 completed NCT04865536 completed NCT06192160 not yet recruiting
Tedizolid		50*S* ribosomal subunit	IIa NCT05534750 recruiting NCT01461460 completed NCT00876655 completed NCT01271998 completed NCT01623401 completed NCT00983255 completed NCT01496677 completed NCT01442831 completed NCT01156077 completed NCT00671814 completed NCT00671359 completed
GSK3036656		Leucyl-tRNA synthase (interfering with proteosynthesis)	IIa NCT03557281 completed with results NCT03075410 completed with results NCT05382312 recruiting NCT06114628 recruiting
Sudapyridine (WX-081)		ATP synthase (involved in energy metabolism)	IIa/IIb NCT06117514 completed NCT05824871 enrolling by invitation NCT04608955 completed
TBAJ-876		ATP synthase	Ib/IIa NCT05526911 completed NCT04493671 completed NCT06058299 recruiting
TBAJ-587		ATP synthase	Ia/Ib NCT04890535 completed
Telacebec (Q203)		QcrB subunit of cytochrome *bcc* complex (involved in energy metabolism)	IIa NCT02858973 completed NCT02530710 completed NCT03563599 completed
SPR720		DNA gyrase GyrB (DNA synthesis and reparation)	II NCT05955586 completed NCT03796910 completed NCT05966688 completed (clinical trials for the treatment of non-tuberculous mycobacterial infections) NCT05496374 recruiting NCT04553406 terminated with results
GSK2556286		cAMP-mediated inhibition of cholesterol catabolism	Ia/Ib NCT04472897 recruiting
Pyrifazimine (TBI-166)		Intracelullar redox cycling-release of oxygen species	IIa NCT04670120 unknown status
Sanfetrinem		L,D-transpeptidases (cell wall biosynthesis)	II NCT05388448 recruiting
OTB-658		50*S* ribosomal subunit	Preclinical GLP toxicology studies
TB47		QcrB subunit of cytochrome *bcc* complex	Preclinical GLP toxicology studies
GSK839		Tryptophan synthase (essential enzyme for *Mtb* survival in macrophage)	Preclinical GLP toxicology studies
MBX-4888A		16*S* ribosomal RNA	Preclinical GLP toxicology studies

a Current status as of 5-2024; data gained from clinicaltrials.gov.

Anti-TB agents research is a highly active field, encompassing numerous classes of compounds with diverse mechanisms of action. The elucidation of structural and biochemical differences between the bacterial and human species is key for the rational design of new antibacterial compounds that can selectively target mycobacteria^17^. A main feature responsible for mycobacteria's unique properties is their cell wall, which is significantly different from cells in other organisms^18^, becoming the primary target for current drugs and drug candidates. Other targets stem from the differences in proteosynthesis, energy metabolism, and synthesis and repair of nucleic acids^19^. Despite the large number of research papers published each year on the design and synthesis of new anti-TB agents, only a small number of these molecules have the necessary drug-like properties and meet sufficient *in vivo* activity. Furthermore, only a fraction of qualifying candidates progress to clinical studies.

Current studies devoted to TB treatment from the perspective of developing new drugs are mainly concentrated on new drug regimen proposals and treatment strategies^20^, ^21^, ^22^, or overviews current and potential drugs^23^^,^^24^. This review examines compounds that are currently being investigated in clinical trials or in advanced preclinical development. Individual agents are clustered based on their mechanisms of action on specific cellular structures or enzyme pathways of *Mtb.* All minimum inhibitory concentrations (MIC) and half-maximal inhibitory concentration (IC_50_) values are reported in micromolar (μmol/L) or nanomolar (nmol/L) values, to facilitate comparison. For MIC values, the percentage of inhibition is indicated by a subscript, and the incubation time and the strain evaluated are reported as recorded in the original publication.

## 2 Inhibitors of cell wall component synthesis

**Figure 1:**
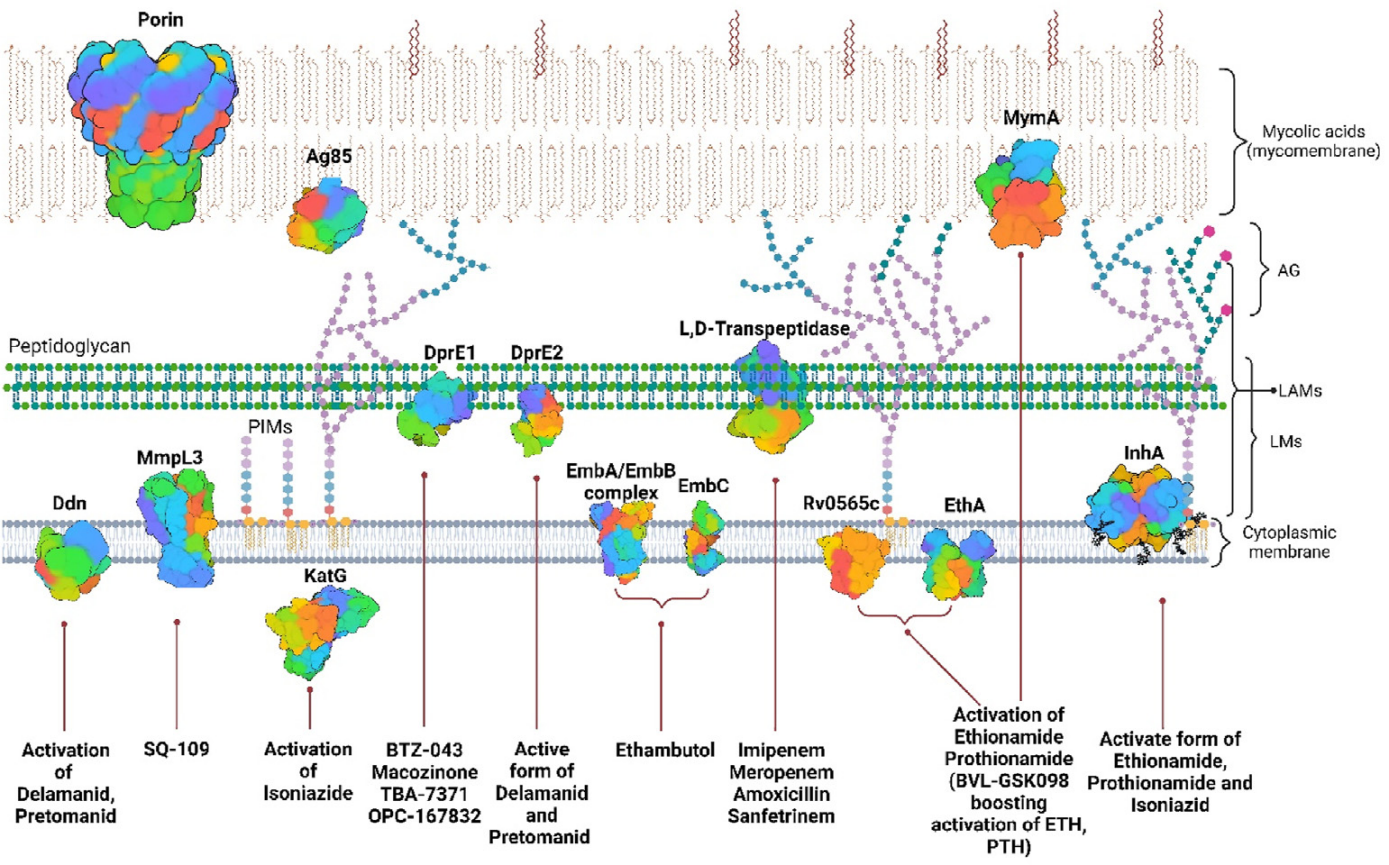
Composition of the mycobacterial envelope and drug targets of known anti-TB drugs. The mycobacterial cell envelope consists of four distinct units: the cytoplasmic membrane, the periplasm, the mycomembrane, and the capsule. Enzymes and transporters within this envelope intricately contribute to the synthesis of cell wall components and overall homeostasis. The cytoplasmic membrane is a glycerophospholipid bilayer incorporating essential molecules such as phosphatidylinositol mannosides (PIMs), lipomannans (LMs), and lipoarabinomannans (LAMs). Molecules derived from PIMs, are pivotal for maintaining cell wall integrity^28^ and immune modulation^29^.

Targeting the mycobacterial cell wall (Fig. 1) biosynthesis has proven to be a highly effective strategy, as exemplified by first-line drugs such as INH^25^ and EMB^26^, as well as several second-line anti-TB agents, including the recently approved delamanid^14^ and pretomanid^15^. The cell wall is an attractive target in *Mtb* as it possesses unique enzymes and transporters involved in cell wall biosynthesis, rendering highly selective antimycobacterial drugs with minimal inter-species toxicity. Another significant advantage lies in the specific localization of the targeted enzymes and transporters within the mycobacterial cell wall, specifically in the periplasm and cytoplasmic membrane. This strategic positioning enhances their accessibility as therapeutic targets compared to those located within the cytoplasm^27^. From this perspective, compounds that interfere with mycobacterial cell wall biosynthesis hold immense promise for developing highly selective anti-TB drugs.

Historically, the first known antibiotics targeting cell wall biosynthesis were the *β*-lactams. However, their effectiveness against *Mtb* was limited due to several factors: the presence of highly potent mycobacterial *β*-lactamases (BlaC, EC: 3.5.2.6)^30^, high expression of efflux pumps^31^, low permeability through mycomembrane of the cell wall^18^^,^^32^^,^^33^, and a slow cell cycle of *Mtb*^34^. Despite these drawbacks, *β*-lactams continue to be used in clinical practice. Carbapenems, a subgroup of *β*-lactam antibiotics, are widely employed for TB treatment in combination with other anti-TB drugs^35^. Their mode of action involves the inhibition of peptidoglycan synthesis. Peptidoglycan, the main component of the periplasm, maintains cell shape. Carbapenems act as inhibitors of l,d-transpeptidases (EC: 2.3.2.-) during cell division. The l,d-transpeptidase is essential for peptidoglycan synthesis by forming a crosslink between peptides and glycan chains formed from *N*-acetylglucosamines and *N*-acetylmuramic acids^36^, ^37^, ^38^. *β*-Lactam antibiotics are listed in the group C according to the WHO classification of anti-TB drugs for the treatment of MDR-TB in prolonged regimens (Table 2). Examples include imipenem (carbapenem) combined with the human dehydropeptidase inhibitor cilastatin, meropenem (carbapenem), and amoxicillin (aminopenicillin) combined with the *β*-lactamase inhibitor clavulanate^39^. Sanfetrinem cilexetil, a tricyclic carbapenem currently undergoing phase 2 clinical trials (Table 3), demonstrated promising intracellular activity with a MIC_50_ of 0.85 μmol/L against *Mtb* H37Rv in monocytes isolated from the blood of an acute monocytic leukemia patient (THP-1 cell line) after five days^40^. Its oral administration potential provides it with an advantage over meropenem^41^.

**Figure 2:**
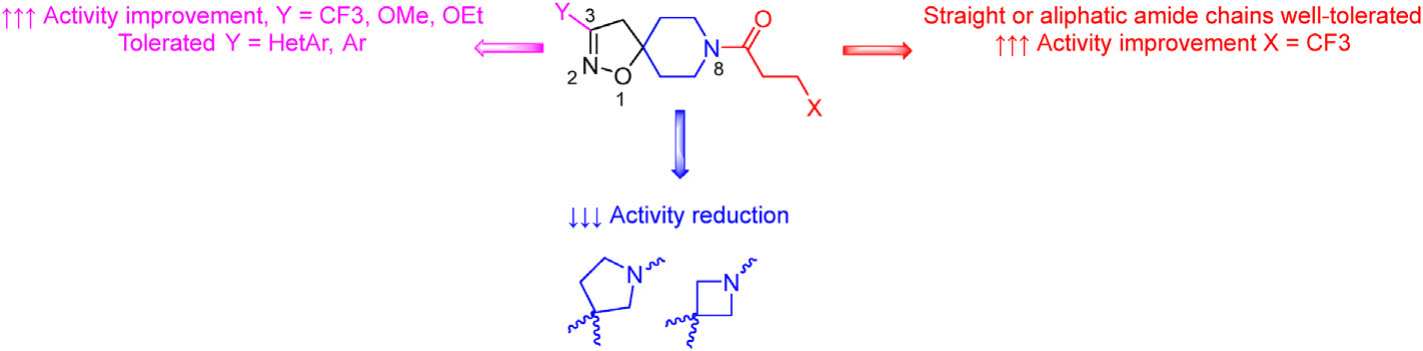
Structure and insight into structure–activity relationships of BVL-GSK098.

Enoyl-[acyl-carrier-protein] reductase (InhA, EC: 1.3.1.9) serves as a target for the activated forms of INH, ethionamide (ETH), and prothionamide (PTH). InhA plays a pivotal role in the mycobacterial type II fatty acid synthase (FA*S* II) system, which is essential for the biosynthesis of mycolic acids^42^^,^^43^. These agents function as prodrugs: INH is activated by the catalase-peroxidase enzyme (KatG, EC: 1.11.1.21)^44^, while ETH and PTH are activated by FAD-containing monooxygenases EthA (EC: 1.14.13.-), MymA (EC: 1.14.13.-), and Rv0565c (EC: 1.14.-.-)^45^. The active forms of these drugs share a common MOA; they form covalent adducts with the NAD cofactor, acting as tight-binding competitive inhibitors of InhA and thereby blocking mycolic acid biosynthesis^42^^,^^43^. The most prevalent cause of resistance to INH is the mutation of the KatG enzyme, preventing the activation of the prodrug and compromising its efficacy. The S315T variant can be found in 94% of IR-TB clinical isolates^46^. Conversely, resistance to ETH or PTH commonly arises from mutations in the promoter region of InhA, leading to its overexpression^47^. BVL-GSK098, currently in phase 2 clinical trials, is endowed with a unique MOA compared to other anti-TB drugs. BVL-GSK098 acts as a transcriptional regulator, stimulating the expression of MymA, a key enzyme activator for ETH and PTH. This stimulation enhances the activity of ETH and PTH, presenting a promising avenue for TB treatment^40^^,^^48^^,^^49^. From the perspective of structure–activity relationships, BVL-GSK098 allows several modifications that maintain high activity. The structure of BVL-GSK098 can be divided into three regions of potential medicinal-chemistry interest: the amidic, central spirocycle, and substituent in the 3-position of the spirocycle (Fig. 2). Molecule tolerates unbranched or branched aliphatic chains with fluorine being most advantageous. Specifically, 4,4,4-trifluorobutanoyl has shown to be the most active. Introducing an aromatic or saturated cycle into the side chain led to a decrease in activity. Replacing the piperidine ring of the central 1-oxa-2,8-diazaspiro[4.5]dec-2-ene moiety with pyrrolidine or azetidine reduced the activity. Various substituents were also tolerated in the 3-position, highlighting ethoxy, methoxy, and trifluoromethyl substituents. However, derivatives with heteroaromatic or phenyl moieties in the 3-position also showed good activity^50^^,^^51^.

**Figure 3:**
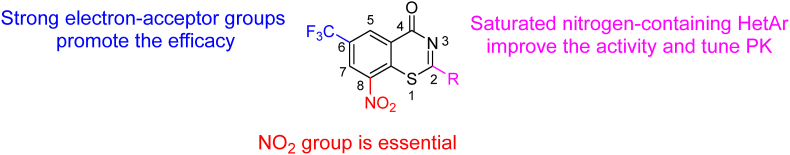
General structure of benzothiazinone and insight into their structure–activity relationships.

Decaprenylphosphoryl-*β*-d-ribose oxidase (DprE1, EC: 1.1.98.3), a part of DprE1-DrpE2 complex, was first described in 2009^52^. DprE1 is a periplasmic flavoenzyme that catalyzes the first step of epimerization of decaprenylphosphoryl-d-ribose (DPR) into decaprenylphosphoryl-d-arabinose (DPA), providing the source of arabinose. Arabinose is an essential monosaccharide for the synthesis of arabinans, lipoarabinomannans (LAMs) and arabinogalactans (AG). EMB targets this enzymatic pathway *via* arabinosyl transferases (EmbA-C, EC:2.4.2.-) inhibition^26^. DprE1 is located in the periplasm, allowing targeting by the xenobiotics^53^. Among them, benzothiazinones received particular attention, represented by macozinone^54^ (*Mtb* H_37_R_v_ MIC_99_ = 0.65 nmol/L 7 days)^55^ and BTZ-043^56^ (*Mtb* H_37_R_v_ MIC_99_ = 2.3 nmol/L 7 days)^55^ undergoing phase 1 and phase 2 clinical trials, respectively^40^. Benzothiazinones are prodrugs that act as suicide inhibitors of DprE1; they are activated *in situ* in the catalytic pocket of DprE1 by a reduced form of the cofactor dihydroflavine-adenine dinucleotide (FADH_2_) *via* the reduction of the nitro group to its nitroso form, which subsequently forms a covalent bond with C387 of DprE1^57^. The MOA suggests that the nitro group in position 8 of the benzothiazinone scaffold is essential for the activity, implying limited possibilities for further structural modifications (Fig. 3). The strong electron acceptor substituents at position 6 and the unsubstituted positions 5 and 7 are pivotal for the activity. Position 2 of the core benzothiazinone moiety is the only allowing modifications. In this position, substituted saturated nitrogen heterocycles are preferred, providing the possibility of pharmacokinetic properties tunning^55^^,^^58^^,^^59^.

**Figure 4:**
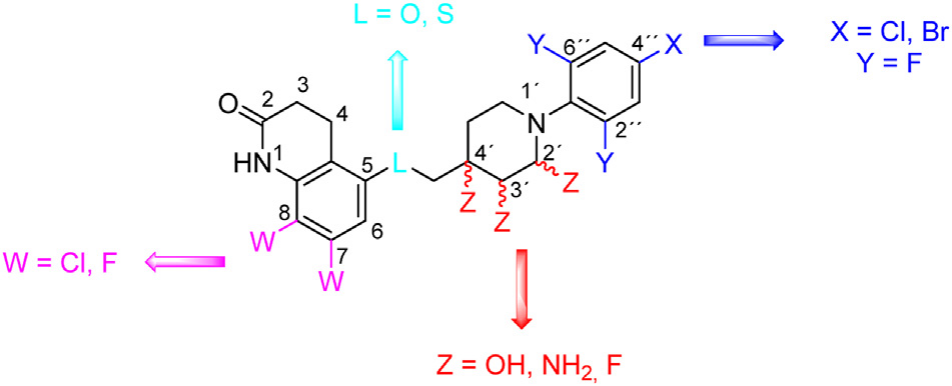
Tolerated structural modifications of OPC-167832.

3,4-Dihydrocarbostyril drug OPC-167832 (*Mtb* H_37_R_v_ MIC_99_ = 0.001 μmol/L) and 4-azaindole derivative TBA-7371 (*Mtb* H_37_R_v_ MIC_80_ = 1.56 μmol/L 5 days)^60^ are other representatives from DprE1 inhibitors, currently undergoing phase 2 of clinical trials^40^. Notably, OPC-167832 is a non-covalent inhibitor, also acting in the catalytic pocket of DprE1, but unlike benzothiazinones, interaction with C387 is not crucial for its activity. Hence, the mutation in this position yields only moderate resistance. Highly OPC-167832 resistant strains rendered mutation at position Y314, being the cause of activity loss^61^^,^^62^. SAR study to OPC-167832 investigated three regions of the molecule, namely 2(1*H*)-quinolinone scaffold, the piperidine moiety attached to position 5 of the core *via* a methylenoxy linker, and an aromatic moiety attached to the piperidine (Fig. 4). Analogues of the 3,4-dihydrocarbostyril moiety displaying enhanced activity were those that were substituted within positions 7 or 8. The preferred substituents were mainly fluorine and chlorine. Regarding the linker, derivatives-bearing oxygen or sulfur atoms maintained notable activity. Nonetheless, for enhanced metabolic stability, oxygen was preferred, prompting the retention of the methylenoxy linker in subsequent modifications. Piperidine ring modifications in positions 2′, 3′ and 4′ with substituents allowing the formation of hydrogen bonds, specifically amino, fluoro, and hydroxyl groups, all yielded activity improvement. Replacement of the piperidine ring with cyclohexane was tolerated in terms of activity, but the lipophilicity was also increased. The aromatic part attached to position 1′ of piperidine predominantly comprised a phenyl group; however, pyridine-2-yl and quinoline-2-yl were also tolerated. Introducing fluorine in positions 2″ and 6″ of the phenyl ring, or a proton-accepting functional group, conferred activity enhancement. Chloro or bromo substituents were the most potent for position 4′′^61^, ^62^, ^63^.

**Figure 5:**
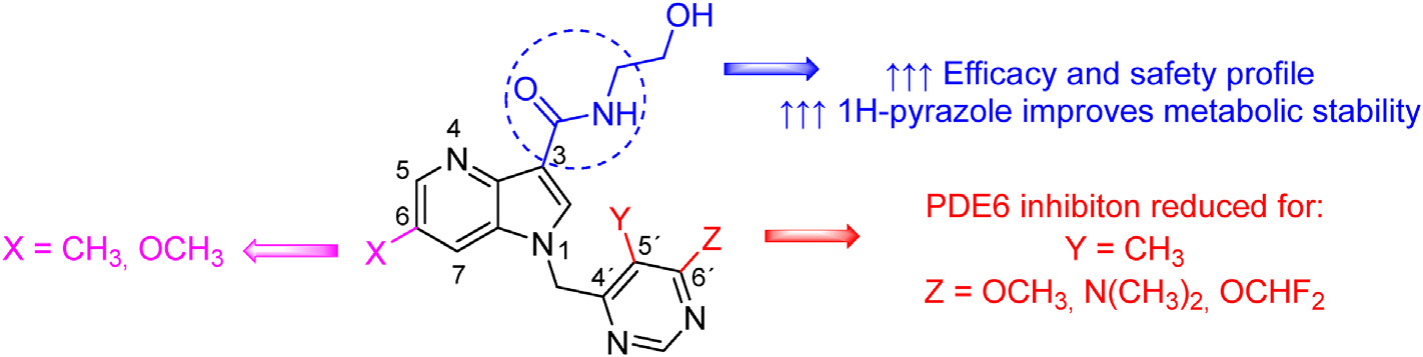
Modification for TBA-7371.

The TBA-7371 mode of action is deemed to be the same as for OPC-167832. The TBA-7371 resistant strain also revealed a mutation in DprE1-Y314 with no cross-resistance to benzothiazinones. Non-covalent interaction with DprE1 was confirmed by mass spectrometry analysis of DprE1 treated with TBA-7371. The pharmacological testing of TBA-7371 revealed inhibition of phosphodiesterase 6 (PDE6, EC: 3.1.4.34) IC_50_ = 4 μmol/L, which may lead to ocular side effects^60^^,^^64^^,^^65^. From a structure–activity relationship perspective, compound TBA-7371 was investigated in three regions; the amide part in position 3 of the 4-azaindole moiety, the heteroaromatic motif substitution attached to the 4-azaindole, and changes in positions 5, 6 and 7 of 4-azaindole (Fig. 5). The nature of the linker attached to *N*-amidic moiety was crucial for the solubility and microsomal stability of the compound. It also affected interaction with PDE6. For all these considerations, 2-hydroxyethyl substituent was the most advantageous. A bioisosteric pyrazole can also replace the amide bond while maintaining activity. Derivatives with nitrogen-containing heteroaromatics or phenyls were prepared to study position 1, revealing that the chosen aromatic moiety did not play a significant role in the activity. However, the substitution of the heteroaromatic moiety in this region significantly affects PDE6 inhibition and solubility. The most preferred heteroaromatic moieties reducing PDE6 inhibition were 6-methoxy-5-methylpyrimidine-4-yl, 6-(dimethylamino)-5-methylpyrimidine-4-yl and 6-(difluoromethoxy)-5-methylpyrimidine-4-yl. Substitutions in positions of 4-azaindole core pointed out to position 6, with a preference for methyl or methoxy substitutions, while a trifluoromethyl group, for instance, yielded in an inactive compound^66^.

Decaprenylphosphoryl-2-keto-*β*-d-erythropentose reductase (DprE2, EC: 1.1.1.333) the second enzyme of DprE1-DprE2 complex, is probably inhibited by active form of pretomanid and delamanid, the most recently approved anti-TB drug, which are prodrugs that are activated by the cytoplasmic enzyme deazaflavin-dependent nitroreductase (Ddn, EC: 1.1.98.-)^67^.

**Figure 6:**
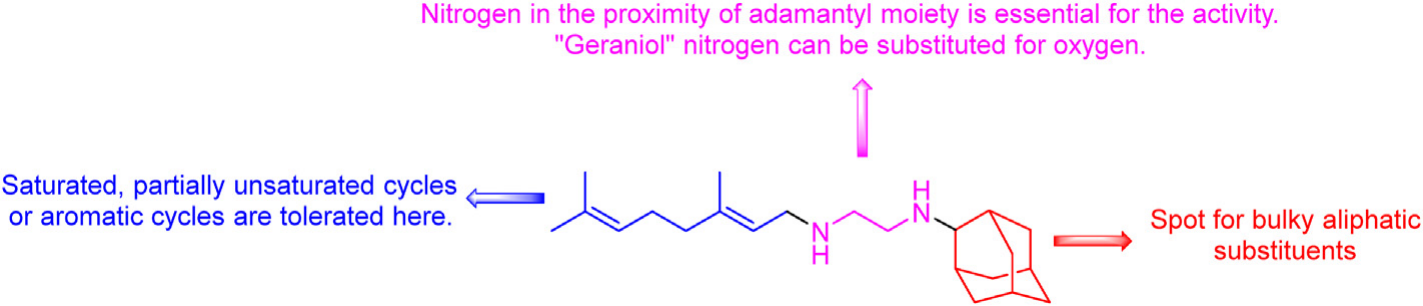
SQ109 and potential structural changes to retain high activity.

Mycobacterial membrane protein large 3 (MmpL3) belongs to the resistance, nodulation and cell division (RND) superfamily of transporters and plays a vital role in many cellular processes such as cell wall biosynthesis, energy metabolism, and presumably iron uptake^68^, ^69^, ^70^. Depletion of MmpL3 is associated with the death of *Mtb*, thus representing a suitable target for potential anti-TB agents. MmpL3 is responsible for transporting trehalose monomycolates (TMM), which is crucial for cell wall synthesis. TMM is synthesized in the cytoplasm from trehalose and mycolic acids. TMM serves as a substrate for mycoloyltransferase AG85 in the biosynthesis of trehalose dimycolates (TDM), a mycomembrane component. TDM, located at the external cell wall layer, plays a key role in immunomodulation during TB pathogenesis^71^. The driving force for TMM transport is the proton cotransport. Inhibition of MmpL3 leads to the accumulation of TMM in the cytoplasm, impairing the cell wall stability, viability, and virulence of *Mtb*^72^. An ethylenediamine MmpL3 inhibitor SQ109 is currently in phase 2 clinical trials, reaching MIC_99_ values of 1.59 μmol/L against *Mtb* H_37_R_v_ and 2.3–3.3 μmol/L against replicating and non-replicating *Mtb*, respectively^40^^,^^73^^,^^74^. SQ109, a transporter inhibitor, has been tested in combinations with standard drugs showing synergism with RIF, INH, and BDQ, increasing the accumulation of the drug in *Mtb*^75^, ^76^, ^77^. SQ109 was identified in a study of EMB analogues. However, a different MOA was subsequently determined, pointing out the inhibition of the MmpL3 transporter^78^. SQ109 acts as a multitarget ligand against MmpL3 and menaquinone (MK) biosynthesis-related enzymes, namely 1,4-dihydroxy-2-naphthoate octaprenyltransferase (MenA EC: 2.5.1.74) and demethylmenaquinone methyltransferase (MenG EC: 2.1.1.163). X-ray analysis of the SQ109 soaked in MmpL3 confirmed hydrophobic interactions between the geraniol appendage and the bulky adamantyl moiety and the hydrogen bonding between the two amino groups with D645. These interactions block the proton channel, thus preventing its cotransport with TMM^79^. Several studies have focused on elucidating the structure–activity relationships of analogues of SQ109. Following structural modifications were considered, specifically the central ethylenediamine linker, and both sides of the molecule either containing geraniol or adamantyl moieties (Fig. 6). It has been confirmed that at least one nitrogen atom must be preserved to retain antimycobacterial activity. The second nitrogen atom can be replaced by oxygen to maintain the activity. The nitrogen attached to adamantyl moiety is vital to keep high *in vitro* activity. Bulky aliphatic substituents are preferred here, with adamant-2-yl moiety representing the drug lead candidate. The geraniol motif is more flexible for changes as saturated or partially unsaturated cycles are tolerated, as well as substituted aromatic rings linked *via* an ethylene linker, where the basic nitrogen is retained. Derivatives with geraniol proved to be the most potent ones^73^^,^^80^.

## 3 Small molecules as disruptors of mycobacterial protein synthesis

Approximately 50% of the cellular energy is used for protein synthesis, an essential process for cellular metabolism and proliferation^81^. This intricate mechanism occurs within ribosomes, complex ribonucleoprotein entities composed of two subunits: a large subunit (LSU), critical for peptide bond formation, and a small subunit (SSU), where the genetic information, encoded in mRNA, is translated into an amino acid sequence. The structural integrity of prokaryotic ribosomes, observed in gram-negative, gram-positive bacteria, and mycobacteria, is largely conserved across species. Subtle interspecies distinctions can be detected by a closer insight into the architecture of individual ribosomal subunits. The small 30S subunit shows minor interspecies structural differences; it is composed of 21 ribosomal proteins and 16S rRNA. In contrast, the 50*S* LSU displays more significant variability, encompassing differences in the number of ribosomal proteins (34 in *Mtb*) and the structural configuration of 23S and 5*S* rRNAs. Notably, mycobacterial ribosomes exhibit prominent disparities in the structure of LSU's 23S rRNA, particularly in the helices of domains I and II, as well as a significantly elongated helix 54a in domain III^82^, ^83^, ^84^.

Streptomycin emerged as the pioneering drug for TB treatment in 1947, but monotherapy led to swift resistance development. Currently, streptomycin remains within Group C to treat MDR-TB, alongside other aminoglycosides such as amikacin and kanamycin (Table 2). The MOA of these aminoglycosides involves binding to 16S rRNA helix 44^85^^,^^86^. Capreomycin is another anti-TB agent from the aminoglycoside class, but its MOA accounts interaction with both subunits of the 70S ribosome^87^. LNZ, a protein synthesis inhibitor, impedes the formation of the 70*S* initiation complex by binding to the 23S rRNA of LSU^88^. In contrast, RIF acts as an inhibitor of DNA-dependent RNA polymerase (EC: 2.7.7.6), hindering RNA synthesis by blocking RNA elongation^89^. Remarkably, this group of protein synthesis inhibitors, excluding RIF, is associated with severe adverse effects including: nephrotoxicity, ototoxicity, and neurotoxicity for aminoglycosides, as well as thrombocytopenia, optic neuropathy, and peripheral neuropathy for LNZ^88^^,^^90^.

**Figure 7:**
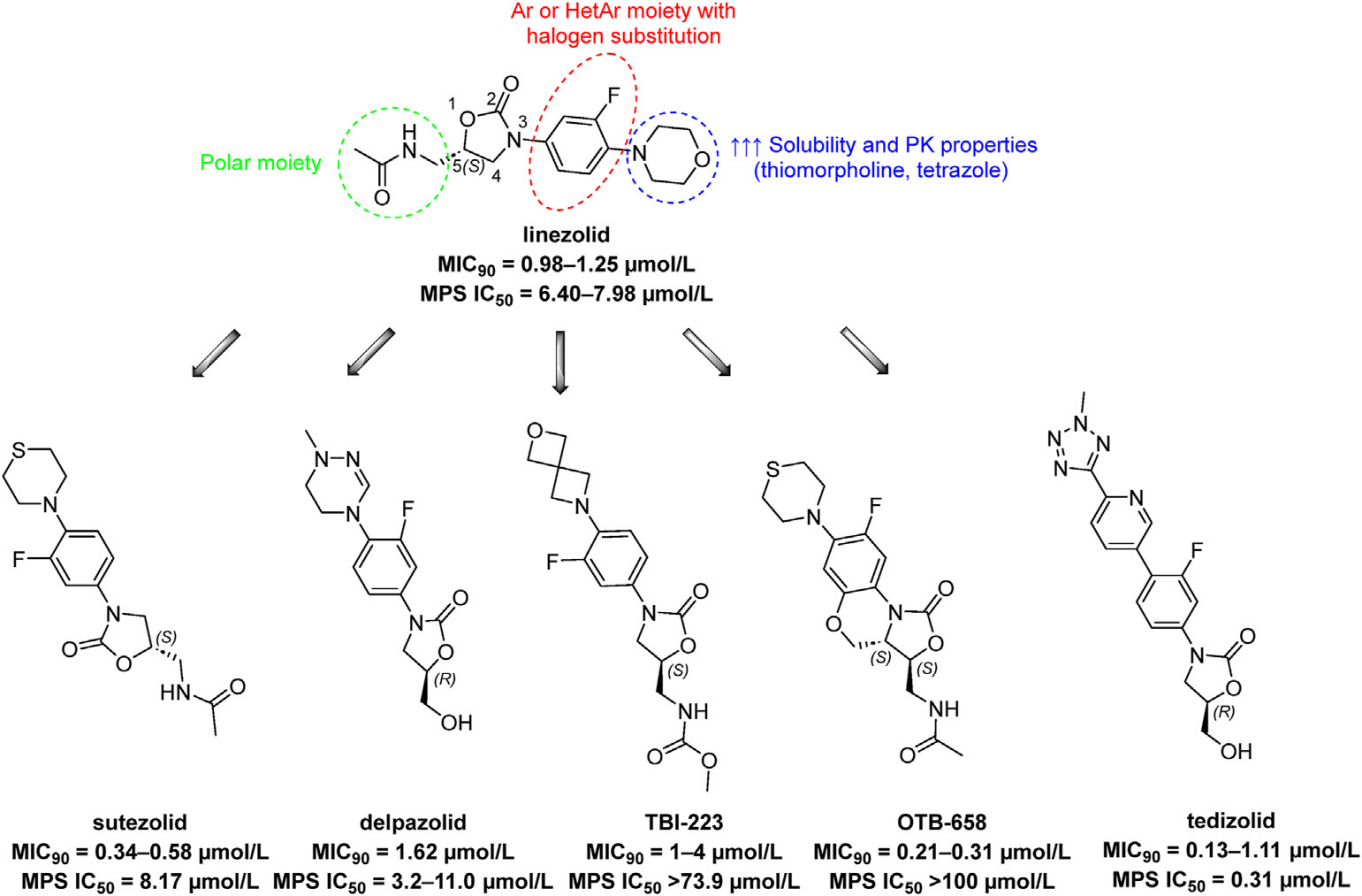
Anti-Tb agents from oxazolidinone family with highlighted regions of the molecule essential for the activity and ADME/PK properties. Examples of clinical candidates are displayed.

Oxazolidinone compounds represent a prominent class of protein synthesis inhibitors, currently under investigation in clinical trials, inspired by the prototype drug LNZ (*Mtb* H_37_R_v_ MIC_90_ = 0.981–1.245 μmol/L 7 days, Fig. 7)^91^. LNZ inhibits cellular proliferation *via* disruption of protein synthesis by binding at the 23S rRNA, preventing the formation of a ribosome complex with *N*-formylmethionyl-tRNA. Clinically, LNZ was approved for the treatment of complicated skin infections and severe pneumonia caused by Gram-positive bacteria. LNZ shows antimycobacterial activity against the *Mtb in vitro,* including drug-resistant clinical isolates. LNZ is extensively used within the realm of MDR-TB therapy, as part of group A in prolonged regimen treatments. However, its usage is conveyed by deleterious side effects, including anemia, and peripheral neuropathy, often necessitating dose reduction or discontinuation^92^^,^^93^. Extensive efforts are under way in the oxazolidinone drug discovery field, to mitigate these adverse effects^94^, ^95^, ^96^, ^97^ The mechanisms responsible for oxazolidinone's toxicity include the inhibition of monoamine oxidases and mitochondrial protein synthesis (MPS), with LNZ MP*S* IC_50_ established at 6.40–7.98 μmol/L^98^^,^^99^. A side effects of long-term LNZ treatment caused by MP*S* inhibition is dose-dependent, reversible bone marrow suppression^100^.

From the medicinal chemistry perspective, all oxazolidinones must preserve an aryl in position 3 and a polar substituent in position 5 attached *via* a methylene linker. The conservation of the spatial arrangement in position 5, which corresponds to the (*S*)-configuration in the LNZ, is crucial for their activity. Substitution of the aromatic moiety influences both activity and, notably, pharmacokinetic properties and solubility. The introduction of fluorine into the phenyl ring is beneficial for activity, while solubility and pharmackokinetics are modulated by the introduction of basic nitrogen in the form of morpholine or thiomorpholine, or the presence of acidic moiety like tetrazole (Fig. 7)^96^^,^^101^^,^^102^.

A subtle modification in the structure, such as replacing morpholine with thiomorpholine in sutezolid (*Mtb* H_37_R_v_ MIC_90_ = 0.34–0.58 μmol/L 7 days, MP*S* IC_50_ = 8.17 μmol/L; Fig. 7), resulted in a slight improvement in the safety profile and a modest increase in antimycobacterial activity^91^^,^^103^^,^^104^. Delpazolid (*Mtb* H_37_R_v_ MIC_90_ = 1.62 μmol/L 7 days, MP*S* IC_50_ = 3.2–11.0 μmol/L; Fig. 7)^99^^,^^105^ is another oxazolidinone currently investigated in phase 2 clinical trials. Replacement of morpholine in LNZ with a cyclic amidrazone, and the substitution of the amide moiety for alcohol enhanced the solubility in delpazolid while concomitantly boosting the safety profile and maintaining efficacy against *Mtb*^106^. The amide fragment in LNZ was replaced by methyl carbamate and the morpholine by spiromorpholine to propose TBI-223 (*Mtb* unspecified strain MIC = 1–4 μmol/L, MP*S* IC_50_ > 73.9 μmol/L; Fig. 7)^23^^,^^99^. These structural changes substantially reduced mammalian MP*S* inhibition, thereby improving the safety profile^107^. OTB-658 (*Mtb* H_37_R_v_ MIC_90_ = 0.21–0.30 μmol/L 7 days, MP*S* IC_50_ > 100 μmol/L; Fig. 7) is an oxazolidinone derivative with *in vitro* potency superior to LNZ and sutezolid. Introducing an additional cycle in the structure led to a conformational constraint, enhancing activity and safety profiles^91^^,^^103^. The most recent oxazolidinone to enter phase 2 of the clinical trial as an anti-TB agent was tedizolid (Fig. 7). This drug, also known as Sivextro, was originally approved by the US Food and Drug Administration (FDA) in 2014 for the treatment of acute bacterial skin infections. The antimicrobial activity of tedizolid was evaluated across 120 DS-TB and DR-TB strains, exhibiting MIC_90_ values in the range of 0.13–1.11 μmol/L. Unfortunately, tedizolid exhibited a higher inhibition of MP*S* with IC_50_ = 0.31 μmol/L^98^^,^^108^.

**Figure 8:**
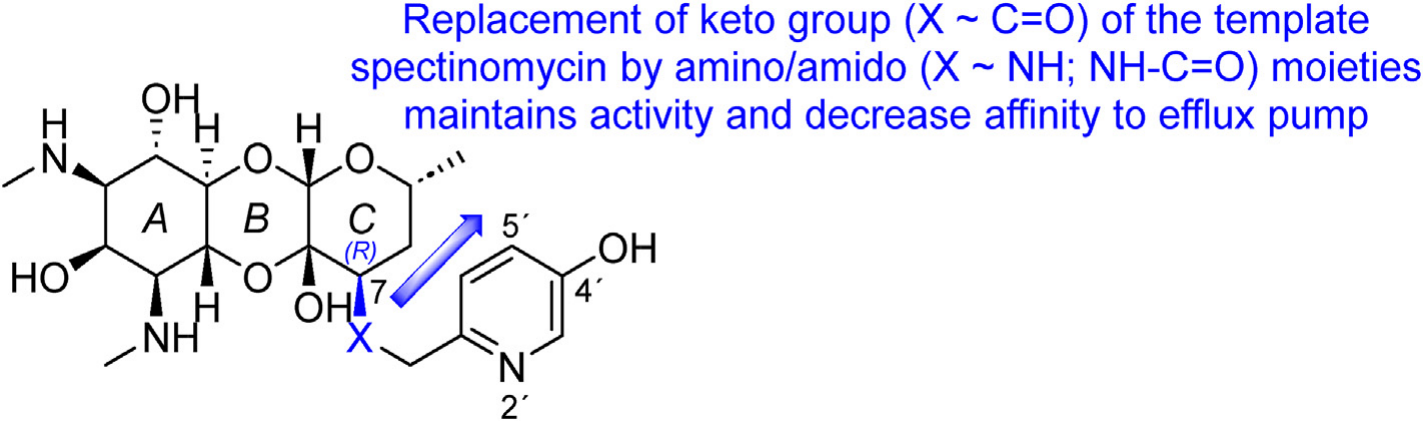
Structure–activity relationships of spectinamides.

Spectinamides (Fig. 8) constitute a semisynthetic group of spectinomycin derivatives. In contrast to structurally analogous aminoglycosides, spectinomycins exhibit minimal adverse effects (ototoxicity and nephrotoxicity have not been observed in this class). Nonetheless, their efficacy against tubercular strains remains limited, primarily attributed to the mycobacterial efflux pump Rv1258c activity. This fact prompted an investigation into the potential structural modification of spectinomycin. Spectinomycin, a tricyclic aminoglycoside comprising actinamine (ring A) and actinospectose (ring C), connected *via β*-glycosidic and hemiketal linkages to form ring B, imposes a challenge for structural alteration due to structural complexity. Model predictions suggested that substituting the keto group in position 7 with an *R*-amine or amide moiety could provide a viable avenue for modification, offering opportunities for further derivatization to attenuate its affinity for the efflux pump. Modifying the amino group with acetamide alternatives emerged as a promising strategy. Among the derivatives synthesized, the (*tert*-butylamino)acetamide derivative displayed superior ribosomal efficacy, albeit with poor whole-cell activity. Subsequent modification with pyridine-2-yl or thiazol-4-yl acetamide groups demonstrated enhanced efficacy. Introduction of halogens in positions 4′ or 5′ of the pyridine moiety further augmented activity, yielding spectinamide-1599 (with a 5-chloropyridin-2-yl moiety, *Mtb* H_37_R_v_ MIC_90_ = 1.64 μmol/L 7 days). Replacement of the chlorine in position 5′ with a hydroxyl group resulted in a remarkable increase in *in vivo* efficacy, as demonstrated in an infected mice model. Spectinamide-1810 (bearing 5-hydroxypyridin-2-yl moiety), known as MBX-4488A, also displayed intriguing activity (*Mtb* H_37_R_v_ MIC_90_ = 3.41 μmol/L 7 days, Table 3). These modifications have also augmented ribosome inhibition through hydrogen bonding within helix 34 of the 16S ribosomal RNA^109^, ^110^, ^111^, ^112^, ^113^.

**Figure 9:**
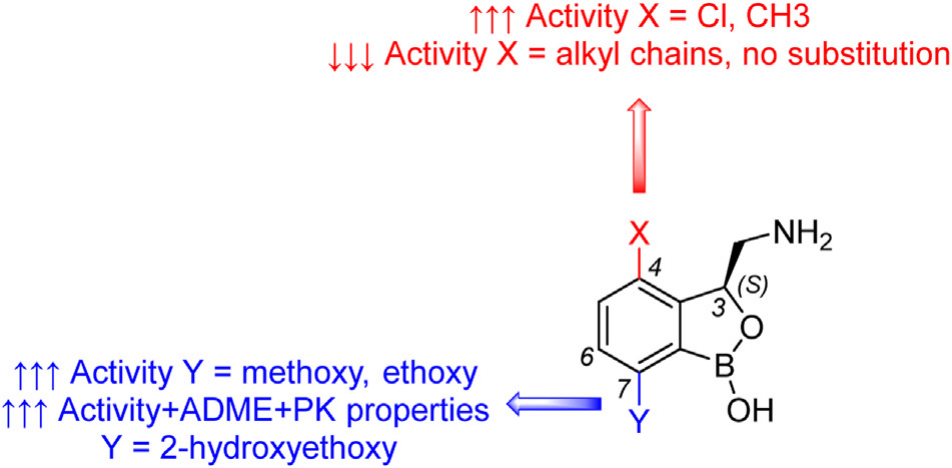
Insight into structure–activity relationships of benzoxaborole family.

GSK3036656 (*Mtb* H_37_R_v_ MIC_95_ = 0.08 μmol/L 14 days, Table 3) represents a groundbreaking advancement as the first boron-based anti-TB agent undergoing clinical trials. This compound belongs to a novel chemical class of 3-aminomethyl-4-halogen benzoxaboroles, exerting inhibitory effects on leucyl-tRNA synthetase (LeuRS, EC: 6.1.1.4). GSK3036656 exhibits remarkable selectivity for *Mtb*, displaying negligible activity against a spectrum of common bacteria and mammalian cell lines^114^, ^115^, ^116^. From a medicinal chemistry standpoint, three substitutions on the benzoxaborole scaffold at positions 3, 4, and 7 are pivotal (Fig. 9). Substitution in position 3 with an aminomethyl group has proven advantageous, with activity attributed to the (*S*)-enantiomer. Position 4 prefers halogen or methyl substitution, whereas longer alkyl chains or unoccupied position led to analogues with reduced activity. Introducing a methoxy or ethoxy group in position 7 has been beneficial; the most valuable turned out to be a 2-hydroxyethoxy appendage that improves ADME and PK properties. Substituent switch from position 7 to 6 significantly diminished the anti-TB activity^114^, ^115^, ^116^, ^117^.

## 4 Chemical drugs interfering with energy metabolism

**Figure 10:**
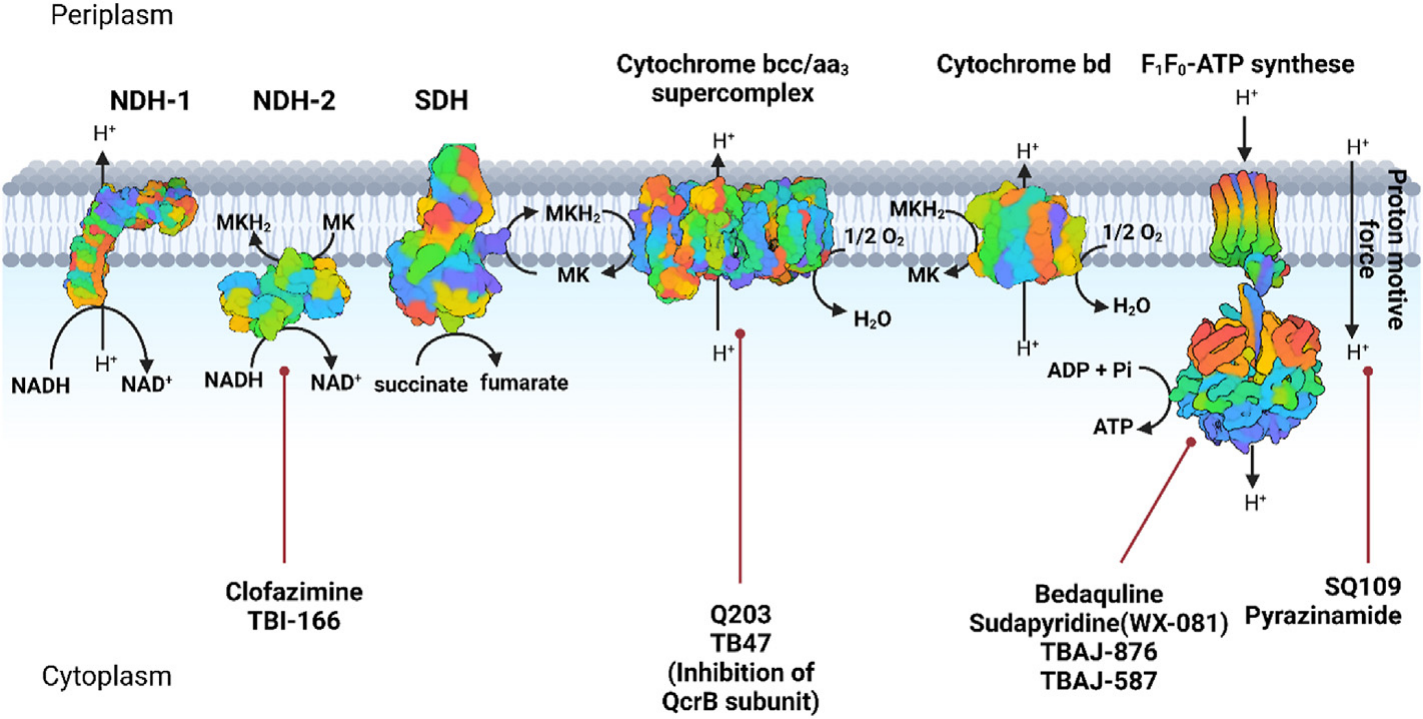
The mycobacterial electron transport chain, and synthesis of ATP are outlined above. Each drug is paired with its target by red line. NADH:quinone oxidoreductases (NDH-1, NDH-2) and succinate dehydrogenase (SDH) function as electron transporters, facilitating the transfer of electrons from NADH and succinate to menaquinone (MK). In its reduced form, menaquinol (MKH2) serves as an electron carrier within the electron transport chain. MKH2 oxidizes back to MK through two pathways: the cytochrome *bcc*/*aa3* supercomplex and cytochrome bd. This oxidation process is coupled with the transfer of electrons to the terminal electron acceptor, oxygen. During the electron transport chain, protons are translocated across the membrane, forming a proton motive force (PMF).

*Mtb* is an obligate aerobic bacterium that relies on respiration and other alternative sources (such as fumarate and nitrate in low-oxygen environments) to generate the energy necessary for maintaining homeostasis. Under aerobic conditions, *Mtb* produces sufficient energy for growth through a series of enzymatic reactions, including the electron transport chain (ETC) and the oxidative phosphorylation system (Fig. 10). Mycobacteria respiration comprises the oxidation of electron donor molecules (*e.g.*, NADH) derived from the catabolism of organic substrates, where oxygen serves as the final electron acceptor^118^. The mycobacterial proteome contains numerous enzymes participants in the ETC, utilizing two alternative pathways for terminal oxidation and electron transfer to oxygen. One pathway involves a cytochrome *bcc* complex and an aa_3_-type family of cytochrome *c* oxidase, while the second pathway includes a cytochrome *bd*-type quinol oxidase. *Mtb* accumulates energy in two forms: adenosine triphosphate (ATP), acting as an energy carrier and store, and the proton motive force (PMF), which serves as the driving force for various metabolic and homeostatic processes, including ATP synthesis. PMF is predominantly harnessed by the membrane enzyme system F_1_F_0_-ATP synthase, and comprises the electric potential Δ*Ψ* (resulting from charge distribution across the membrane) and the chemical transmembrane proton gradient ΔpH^118^, ^119^, ^120^.

Recently, the inhibition of energy metabolism emerged as a promising therapeutic target when BDQ has been marketed (*Mtb* H_37_R_v_ MIC_99_ = 0.054–0.216 μmol/L 21 days)^121^ in 2012^122^. However, its use is accompanied by severe adverse effects, specifically QT interval prolongation due to hERG inhibition, encoding the alpha subunit of the ether-à-go-go-related gene. hERG IC_50_ value of BDQ is 1.6 μmol/L^123^. Shortly after BDQ was introduced into clinical practice, the first cases of resistance emerged, caused by a mutation in the MarR-like transcriptional regulator Rv0678 controlling the expression of the gene encoding mycobacterial membrane protein-small 5- mycobacterial membrane protein large 5 (MmpS5-MmpL5) transporter^124^^,^^125^. Several BDQ analogues have been developed to mitigate side effects and resistance of the parent drug, three of which (TBAJ-587, TBAJ-876, and sudapyridine) are currently in clinical trials (Table 3).

**Figure 11:**
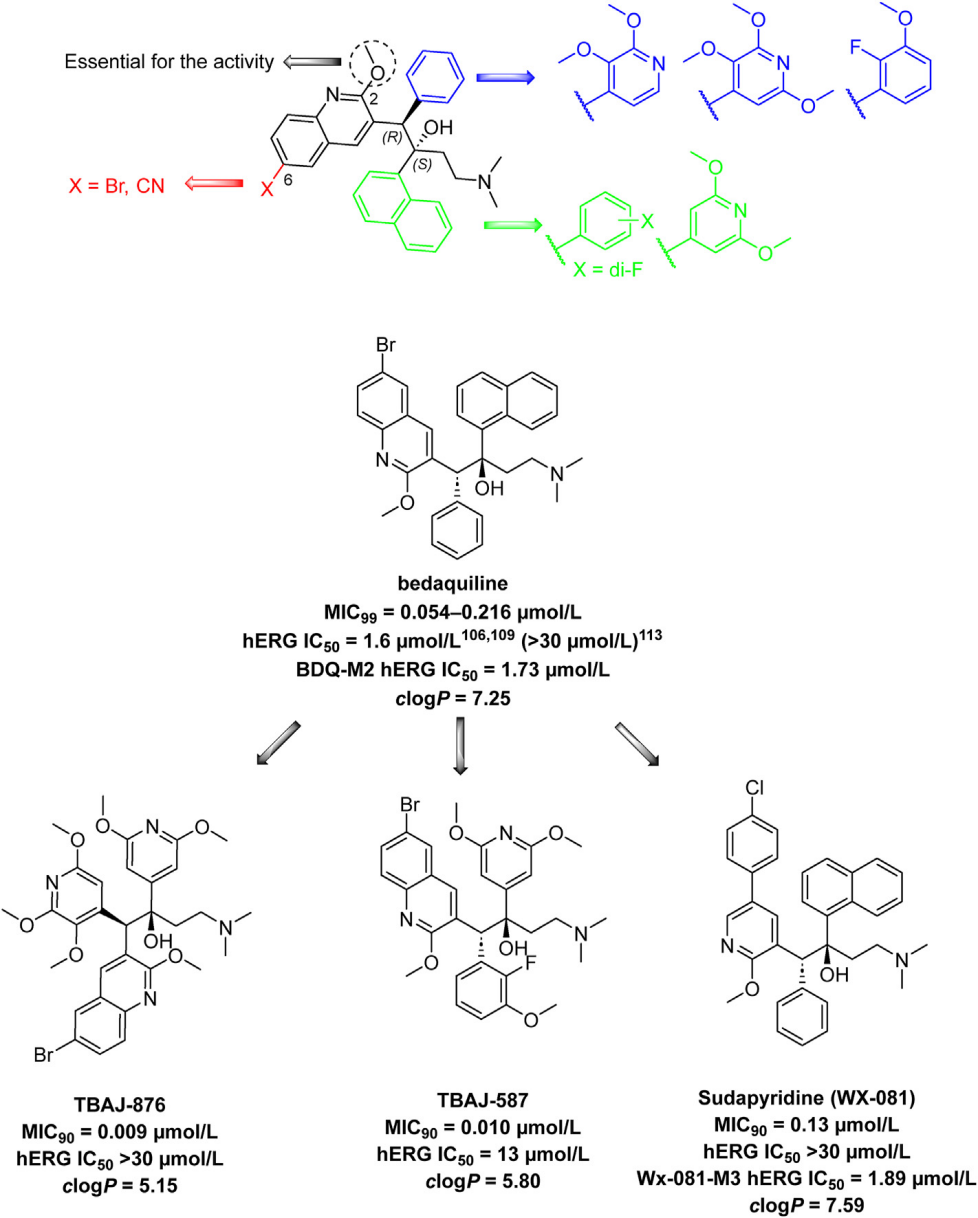
General structural aspects for bedaquiline and other diarylquinolines and biphenyl sudapyridine.

Diarylquinolines, including BDQ (Fig. 11), are currently the largest group of mycobacterial energy metabolism disruptors, acting as selective inhibitors of mycobacterial F_1_F_0_-ATP synthase. BDQ, despite its efficacy, is challenged by its exceptionally high lipophilicity (*c*log*P* = 7.25), leading to a protracted terminal elimination half-life of 5.5 months and significant tissue accumulation. Another critical task in BDQ drug development involves minimizing its affinity for hERG channel, a primary consideration for cardiac safety^126^^,^^127^. The possibilities of structural optimization of BDQ have been the subject of numerous studies, primarily aiming to reduce lipophilicity, which was assumed to be directly linked with cardiotoxicity (Fig. 11). Based on insights into the activity dependence on structural modifications of BDQ, fundamental structural features were retained, namely the methoxy group in the 2-position and the bromine at the 6-position of the quinoline, although further modification also highlighted bromine replacement with a nitrile group^128^^,^^129^. The length of the liker chain between the quinoline moiety and the tertiary amine had to be fixed to preserve high anti-TB efficacy, promoting two potential sites for potential modification, a phenyl and a naphthalen-1-yl moiety. Analogues with a modified phenyl moiety included a variety of heterocyclic substitutions, maintaining activity at a low micromolar to nanomolar range, with 2,3-dimethoxypyridine-4-yl emerging as the most promising^130^. Replacing the naphthalen-1-yl moiety with 2,6-dimethoxypyridine-4-yl was the most effective one^123^^,^^131^. Two compounds codenamed TBAJ-587 and TBAJ-876 (Table 3) are currently under clinical trial evaluation^123^. In the case of TBAJ-587, the strategic modifications implemented the introduction of fluorine at the 2-position and methoxy at 3- position on the phenyl ring, along with the replacement of the naphthalen-1-yl moiety by a 2,6-dimethoxypyridin-4-yl group. These alterations led to a reduction in lipophilicity (*c*log*P* = 5.80) and mitigated cardiotoxicity (hERG IC_50_ = 13 μmol/L) while maintaining potent antimycobacterial activity (*Mtb* H_37_R_v_ MIC_90_ = 9.8 nmol/L). Concerning TBAJ-876, a substitution of the phenyl ring with the more hydrophilic 2,3,6-trimethoxypyridin-4-yl moiety lowered lipophilicity (*c*log*P* = 5.15) and remarkably improved safety (hERG IC_50_ > 30 μmol/L) while retaining robust antimycobacterial activity (*Mtb* H_37_R_v_ MIC_90_ = 9.1 nmol/L). Notably, the impact of the Rv0678 mutation on antimycobacterial activity in TBAJ-876 and TBAJ-587 was lower than BDQ, rendering them potential substitutes for BDQ in TB therapy^123^^,^^132^^,^^133^.

Pyridine-based BDQ derivatives (Fig. 11) emerged as a new class of anti-TB agents acting as ATP synthase inhibitors. Sudapyridine (WX-081) is a clinical candidate that was discovered from an extensive structure–activity relationships campaign, replacing quinoline with phenylpyridine (Fig. 11). For the phenylpyridine moiety, attachment of phenyl to position 5 of the pyridine proved the most effective. Additionally, the substitution in this position with bulky substituents was tolerated but did not offer significant improvement. Further changes in the *para* position of the phenyl ring led to the identification of the two most effective derivatives bearing trifluoromethoxy group or chlorine. Replacement of naphthalene-1-yl moiety by phenyl aggravated toxicity and diminished the activity. Difluorophenyl derivatives were slightly more active and comparably toxic to naphthalene-1-yl-containing BDQ derivatives; however, their chemical synthesis required hardly available intermediates^134^. Consequently, sudapyridine (WX-081) was identified, bearing 5-(4-chlorophenyl)-2-methoxypyridin-3-yl moiety as a surrogate for BDQ quinoline moiety. Sudapyridine retained a potency comparable to BDQ (*Mtb* H_37_R_v_ MIC_90_ = 0.13 μmol/L 7 days); however, lipophilicity increased *c*log*P* = 7.59, yielding high plasma protein binding. hERG inhibition assays revealed similar values for sudapyridine and BDQ (both reaching hERG IC_50_ > 30 μmol/L). While the authors of this study suggest that sudapyridine exhibits a safer profile than BDQ, the evidence provided is inconclusive. This study identified metabolites that may be potentially responsible for cardiotoxicity, where WX-081-M3 and BDQ-M2 (metabolites of sudapyridine and BDQ, respectively) displayed comparable IC_50_ values (WX-081-M3 hERG IC_50_ = 1.89 μmol/L; BDQ-M2 hERG IC_50_ = 1.73 μmol/L)^134^, ^135^, ^136^. The results of the effect of BDQ on hERG reported in the study by Yao et al.^135^ are inconsistent with other studies^123^^,^^128^.

**Figure 12:**
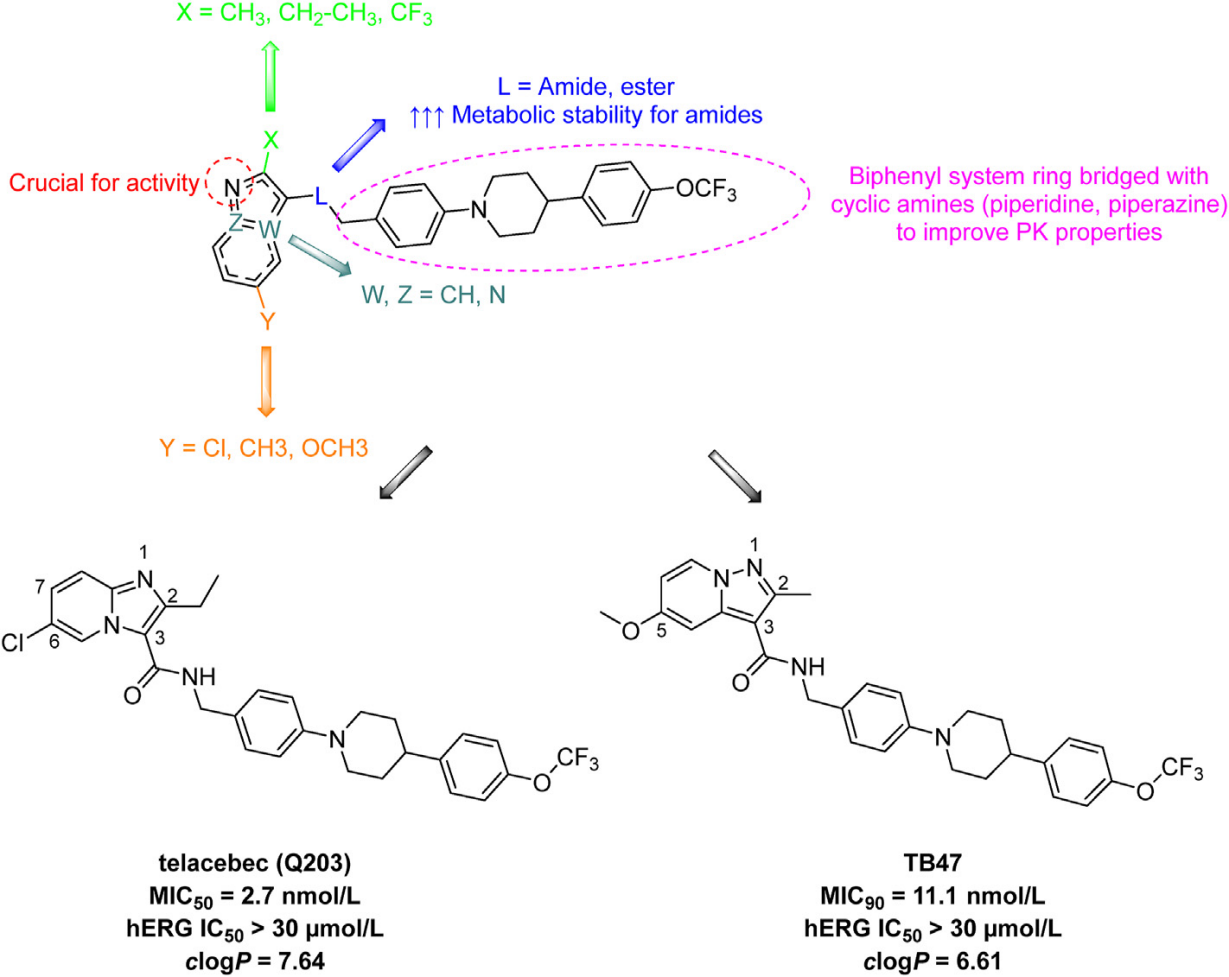
The general structure of imidazopyridine amides and pyrazolopyridine carboxamides as QcrB inhibitors with highlighted key structural fragments and clinical candidate representatives telacebec and TB47.

Imidazopyridine amides are novel anti-TB agents that function as inhibitors of the cytochrome *bcc* complex cytochrome *b* subunit (QcrB EC: 7.1.1.8). The cytochrome *bcc* complex is a crucial component of various enzyme complexes involved in electron transport and the formation of the respiratory ETC. Telacebec (Q203; Fig. 12) is currently in phase 2 clinical trials, exhibiting excellent antimycobacterial activity (*Mtb* H_37_R_v_ MIC_50_ = 2.7 nmol/L 21 days) with minimal hERG affinity (hERG IC_50_ > 30 μmol/L). Despite its high lipophilicity (*c*log*P* = 7.64), telacebec has favorable pharmacokinetic properties (orally available, maximal plasma concentration after oral administration of 2 h and elimination half-life of 23.4 h)^137^^,^^138^. Telacebec was recognized as a part of the optimization study on imidazo[1,2-*a*]pyridine-3-carboxamides, identified through high-throughput screening of compounds against intracellular *Mtb*^138^^,^^139^. Several structural features necessary for activity were pinpointed during the drug modification (Fig. 12). A short alkyl chain or trifluoromethyl group in the 2-position were preferred. Position 3 tolerated carboxamide or ester groups as crucial for the activity, but amidic analogues were favored for higher metabolic stability. Core substitution of imidazo[1,2-*a*]pyridine highlighted positions 6 or 7, with chlorine or methyl being the top-ranked. The amidic side chain had a significant impact on activity and pharmacokinetic properties. Notably, the secondary amidic group had to be preserved to maintain high activity. The biphenyl system ring demonstrated high activity on the one hand, but sub-optimal pharmacokinetic properties on the other hand. Accordingly, the insertion of a cyclic amine (piperazine or piperidine) between two phenyl rings yielded excellent potency^137^^,^^140^^,^^141^. Subsequent studies highlighted the importance of spatial arrangement and electron distribution, particularly discussing the distance between the nitrogen in position 1 of the core moiety and the carboxamide in position 3. Thus, scaffold hopping approach enabled discovery of TB47 built on pyrazolo[2,1-*a*]pyridine-3-carboxamides, with nanomolar activity against *Mtb*^142^, ^143^, ^144^.

Pyrazolopyridine carboxamides are structurally related to imidazopyridine amides, sharing a common target, namely QcrB. Derivatives structurally similar to telecabec emerged as the most potent members of the pyrazolopyridine carboxamide family (Fig. 12). Several pyrazolopyridines have been developed, displaying high activity against *Mtb* H_37_R strains, but only TB47 (Fig. 12) exhibited sustained efficacy against DR-TB strains. TB47 (*c*log*P* = 6.61) demonstrated 10-fold lower lipophilicity compared to telacebec, while maintaining equivalent efficacy (*Mtb* H_37_R_v_ MIC_90_ = 11.1 nmol/L 7 days) and low cardiotoxicity (hERG IC_50_ > 30 μmol/L)^142^^,^^145^^,^^146^.

## 5 Disruptors of DNA replication and reparation

**Figure 13:**
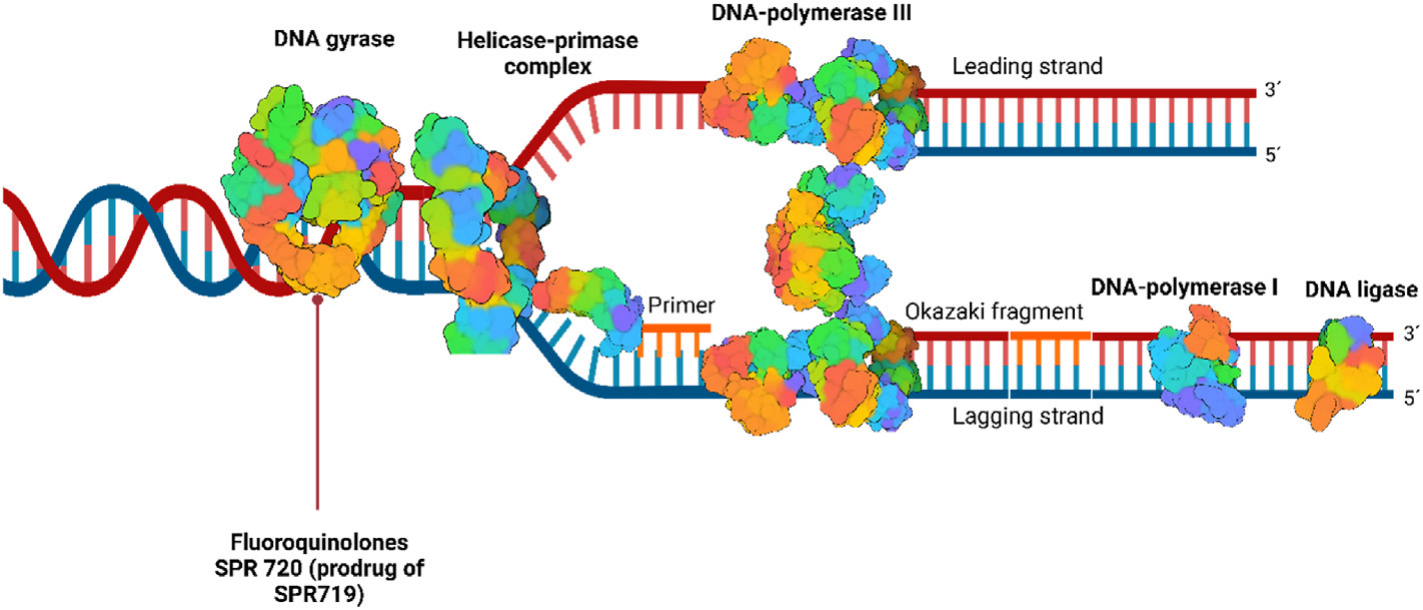
Mycobacterial replication fork as a drug target is a semi-conservative process in which two double strands of new DNA are formed, each carrying one original and one new strand. SPR720 and FQ are paired with its target enzyme by the red line.

The survival and evolution of any living organism depend on its ability to reproduce. The *Mtb* life cycle is a unique process that allows it to survive in hostile environments. *Mtb*'s *genome* spans approximately 4.4 million base pairs, constituting a circular chromosome. Replication initiates with a specific DNA segment known as the replicator, characterized by unique sequences that enable the binding of initiation proteins. This binding event triggers a cascade of processes culminating in the unwinding of the double helix into individual strands at the selected region (oriC sequence)^147^^,^^148^. DNA replication is orchestrated by an array of enzymes and proteins, among which gyrases play a key role by modifying the tertiary structure of DNA through negative super-coiling, thereby facilitating the functioning of helicase. Gyrases facilitate the functioning of the replisome, the molecular machinery upon which replication occurs. Various enzymes rigorously modulate the replisome activity to ensure the precision of DNA replication. This multienzyme complex comprises three catalytic compartments. The first compartment is the helicase–primase complex, responsible for unwinding the DNA strands into a replication fork and synthesizing RNA primers on the lagging strand. The second one is the core complex, harboring DNA polymerase III and facilitating the synthesis of complementary DNA strands, continuously in the case of the leading strand. However, the synthesis of the complementary strands on the lagging strand occurs discontinuously, involving the formation of short DNA fragments known as Okazaki fragments. Primers are later replaced by DNA polymerase I, leading to the formation of a complete double strand. The final step in this complex process involves the sealing of the DNA fragments, a task accomplished by DNA ligase. The clamp loader complex forms the last compartment, which aids the core complex in its functions (Fig. 13). Many enzymes are responsible for DNA replication and are highly conserved across bacteria, including mycobacteria^148^^,^^149^. Within the portfolio of available anti-TB treatments, the FQ family stands as a unique group targeting DNA synthesis and repair through the inhibition of DNA gyrase (EC:5.6.2.2), undermining the DNA replication processes^150^^,^^151^.

**Figure 14:**
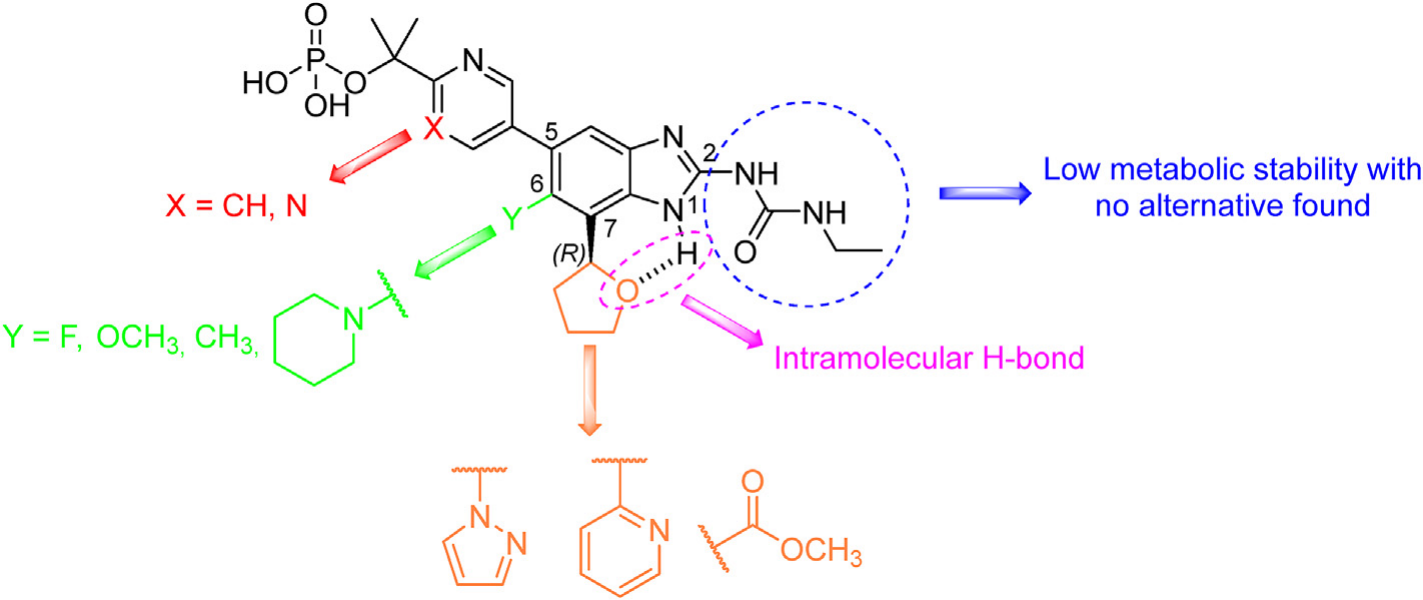
Insight into benzimidazole-urea derivatives structurally related to SPR720.

Benzimidazole urea derivatives are promising DNA gyrase inhibitors, with SPR720 (fobrepodacin) currently undergoing clinical trials. SPR720 (Table 3), a stable phosphate prodrug of SPR719, functions through a mechanism distinct to FQ, binding to the ATP subunit of DNA gyrase. *In vitro* activity of SPR719 (*Mtb* H_37_R_v_ MIC_90_ = 0.28–0.58 μmol/L) is approximately three times lower than moxifloxacin from the FQ family, but SPR720 prodrug and moxifloxacin showed similar efficacy *in vivo*^152^^,^^153^. Benzimidazole urea derivatives were first described as antimicrobial agents active against a broad spectrum of G+ and G-bacteria. Subsequent structural modifications focused on exploring the influence of substitution in positions 2, 5, 6 and 7 of the benzimidazole core in relationship to activity against *S. aureus* and other bacteria (Fig. 14). Ethyl urea in position 2 was found to be metabolically unstable, necessitating its replacement. However, several urea mimics were inactive, except for the carbamoyl analogue which also revealed lower metabolic stability. For this reason, the ethylurea was preserved for sequential structural modifications. Nitrogen-containing heterocyclic compounds were favored in position 5, specifically pyridin-3-yl, pyrimidin-5-yl, and 2-(2-hydroxypropan-2-yl)pyrimidin-5-yl. The heterocyclic nitrogen played a pivotal role in the hydrogen bond formation with R136 of DNA gyrase subunit B (GyrB) from *E. coli* and R144 of GyrB from *S. aureus*. Position 6 tolerated a wide range of substituents; notably, fluorine derivatives exhibited slightly higher activity, suggesting enhanced permeability through the bacterial cell envelope. Position 7 accommodated preferably pyrazol-1-yl or tetrahydrofuran-2-yl [in (*R*)*-*configuration], allowing intramolecular hydrogen bonding with NH of benzimidazole moiety^154^, ^155^, ^156^.

## 6 Anti-TB agents with miscellaneous mechanisms of action

**Figure 15:**
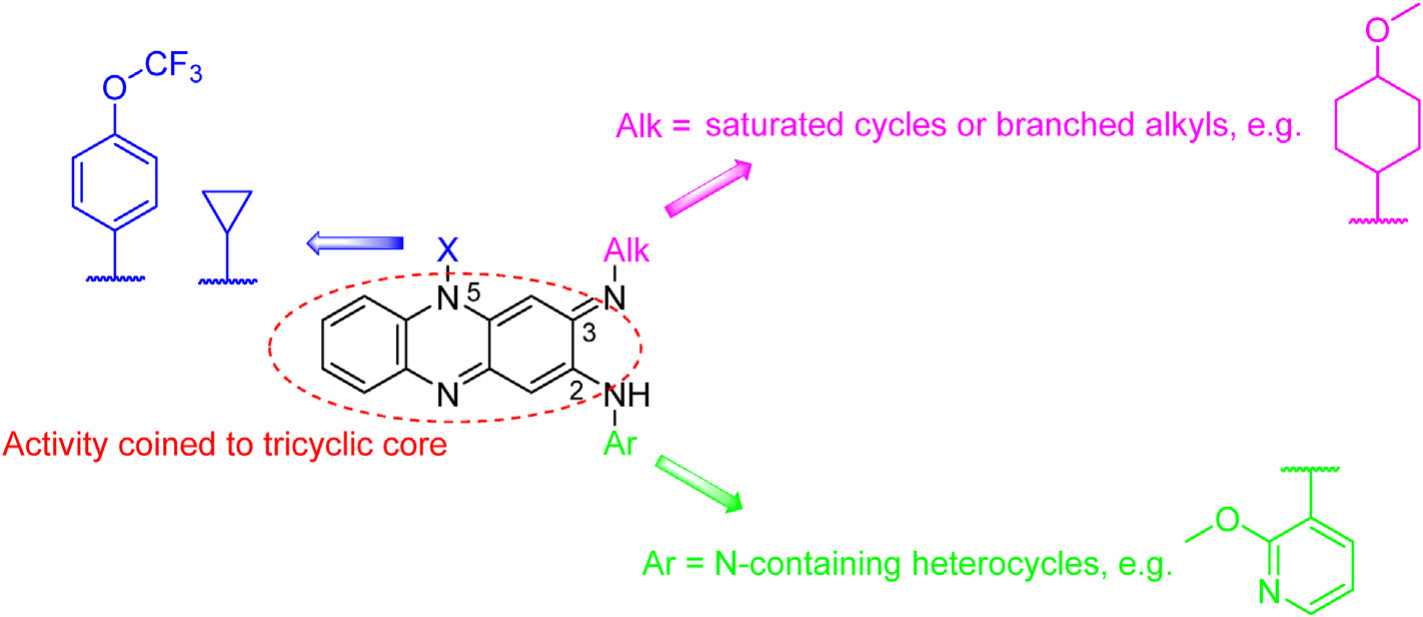
TBI-166 and structure–activity relationships of riminophenazines.

Riminophenazines, a class of antibiotics represented by clofazimine (*Mtb* H_37_R_v_ MIC_90_ = 0.255 μmol/L 7 days (Table 2)^157^, have been investigated for the treatment of infections caused by *Mycobacterium leprae*. Clofazimine was originally developed as a drug against *Mtb,* but it was discontinued due to low *in vivo* activity^158^^,^^159^. The drug was off-labeled as part of a combination regimen to treat DR-TB in Bangladesh^160^, demonstrating good efficacy and reducing the treatment duration. Clofazimine is currently listed in the standardized regimen for DR-TB according to WHO (Table 1) and Group B of the individualized regimen (Table 2). The MOA of riminophenazines is not fully understood; one hypothesis suggests the involvement of the drug in the intracellular redox cycle and the subsequent formation of reactive oxygen species that induce oxidative stress. The second hypothesis assumes the competition of clofazimine with MK for the enzyme type II NADH oxidoreductase (EC: 1.6.5.9), which functions early in the mycobacterial respiratory chain^159^. A major issue of clofazimine administration is associated with adverse side effects, mainly the discoloration of skin tissues caused by the deposition of this highly lipophilic drug (*c*log*P* = 7.7)^157^, ^158^, ^159^, ^160^, ^161^, ^162^. The main goal of current drug development efforts has been to mitigate such undesirable effects, and this was successfully met in TBI-166 (Table 3). *p-*Chlorophenyl in clofazimine was replaced with a 2-methoxypyridin-3-yl moiety linked to the 2-amino group of the riminophenazine scaffold. This modification resulted in a reduction of lipophilicity (*c*log*P* = 6.85) and diminishing skin discoloration while at the same time slightly improving the activity (*Mtb* H_37_R_v_ MIC_90_ = 0.027 μmol/L 7 days)^157^^,^^162^. Possible structural modifications of clofazimine have been studied since its discovery in the 1950's (Fig. 15). Simplification of the tricyclic arrangement by the omission of one benzene ring reduced the efficacy^163^. Aromatic amines were preferred in position 2, with pyridyl derivatives showing more activity than phenyl congeners^157^^,^^162^^,^^164^. Substitution of the imine nitrogen in position 3 with saturate cycles or branched alkyls improved the activity and physicochemical properties compared to aromatic counterparts^157^^,^^162^^,^^164^, ^165^, ^166^, ^167^, ^168^, ^169^. Cyclopropyl or aromatic rings (like in TBI-166) were tolerated in position 5^169^.

GSK2556286 (GSK286, Table 3) is a pyrimidine-2,4-dione derivative discovered *via* the high-throughput screening of compounds, with unknown MOA, against *Mtb*-infected macrophages. Based on the generated drug-resistant mutants, the putative MOA lies in the alteration of cholesterol catabolism. A total 29 spontaneous GSK-286 resistant mutants were generated, out of which 14 possess a mutation in the *Rv1625c* gene, encoding class IIIa membrane-anchored adenylyl cyclase (cya, EC: 4.6.1.1), an important enzyme in the synthesis cyclic adenosine monophosphate (cAMP). cAMP serves as a second messenger, negatively regulating cholesterol utilization. The proposed MOA is consistent with experimental observations; the MIC values in cholesterol medium (with cholesterol being the sole carbon source) were significantly lowered (*Mtb* H_37_R_v_ MIC_90_ = 2.12 μmol/L 7 days) compared to the glucose medium (*Mtb* H_37_R_v_ MIC_90_ >125 μmol/L 7 days)^170^, ^171^, ^172^.

**Figure 16:**
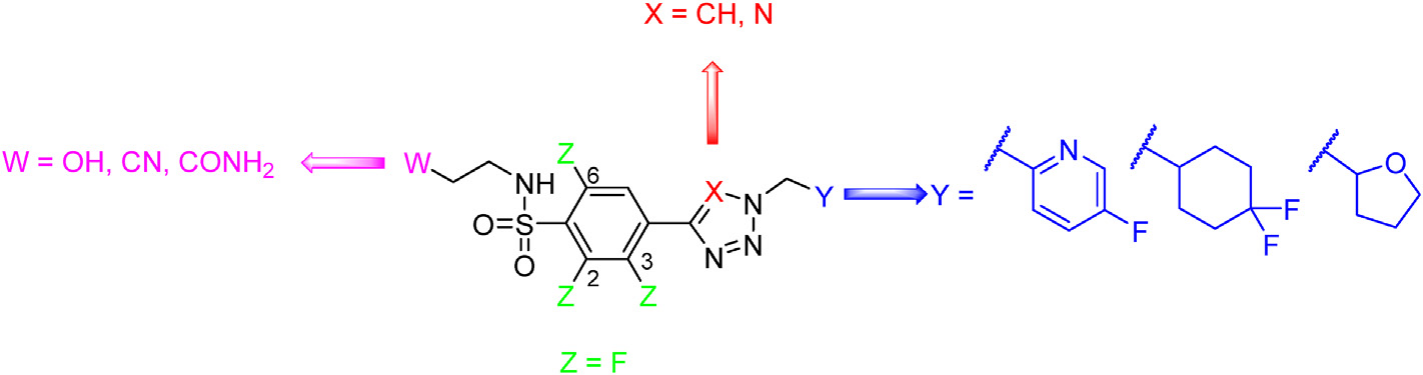
Tolerated changes to GSK839 preserving high anti-TB activity.

Compound GSK839 (Table 3) was patented in 2017 as anti-TB agent inhibiting tryptophan synthase (EC: 4.2.1.20). This enzyme complex is responsible for the last two steps of l-tryptophan biosynthesis, allowing *Mtb* to supply l-tryptophan on its own and thus increasing its ability to survive in diverse environments. GSK839 exhibited an extracellular anti-TB activity value of *Mtb* H_37_R_v_ MIC = 0.07–0.16 μmol/L 7 days^173^. The compound represents four regions of interest that were inspected in detail by medicinal-chemistry principles, specifically the side chain of the amide, the benzenesulfonamide moiety, the 2*H*-tetrazole moiety, and the pyridine-2-yl moiety (Fig. 16). As for the side chain of the amide, the 2-hydroxyethyl chain proved to be the most potent, tolerating also nitrile or primary amide group. Shortening or elongation of the chain led to a decrease in efficacy. The benzenesulfonamide moiety was also examined, proving that the introduction of fluorine in position 3 or two fluorines in positions 2 and 6 of the phenyl ring is beneficial. Replacement of the 2*H*-tetrazole scaffold with 1*H*-1,2,3-triazole maintained the efficacy. Replacement of the pyridine-2-yl moiety with phenyl resulted in a slight increase in activity and lipophilicity. Additionally, the exchange for saturated cycles, specifically 4,4-difluorocyclohexyl and tetrahydropyran-2-yl, was tolerated^173^.

## 7 Conclusions

TB remains a pervasive infectious disease with profound implications for public health and economies worldwide. The COVID-19 pandemic exacerbated the number of infected with TB, making both the diagnosis and treatment of TB less accessible in many countries, thereby increasing the risk of spreading DS-TB and MDR-TB cases^174^, ^175^, ^176^. The WHO emphasized the urgent need for intensified research to identify and develop new drugs and regimens that can lead to treatment shortening^175^. The increasing incidence of drug resistance in TB treatment is a critical indicator of the need for drugs with novel MOA. The important aspect to consider when developing new drugs is to shorten the treatment duration. A recent global surge in anti-TB research efforts led to the discovery of numerous potent molecules annually, yet only a fraction advance to late preclinical phases, and fewer will progress to clinical trials. Indeed, only three new drugs have been approved for the treatment of TB; namely BDQ, delamanid and, most recently, pretomanid, have been marketed. This challenge arises from the rigorous demands of creating compelling and safe drug candidates, with optimal pharmacokinetic and pharmacodynamic profiles, ideally endowed with novel MOA capable of treating DR-TB. The development hurdles are exacerbated by the unique lipophilic cell envelope of the *Mtb*. Besides, *Mtb* is an intracellular pathogen, requiring the drug to act inside the macrophage and being non-toxic to human cells at the same time^177^. The absence of reliable predictive models for mycobacterial cell wall permeation hampers successful biochemical screening. Therefore, whole-cell screening is an important component in developing potential new drugs, but the complexity of target identification and difficulty in hitting optimization are known limiting factors. This review highlighted the current state of anti-TB small molecules, focusing on those in late-stage preclinical development or clinical testing.

Another factor that must be taken into account in the development of potential drugs is the requirement to act on slowly growing or nonreplicating bacteria. Most *in vivo* experiments performed in the development of anti-TB agents focus on determining efficacy in an acute infection model using BALB/c mice as model organisms, which is an excellent model for primary screening. However, it does not provide information on efficacy in the case of LTBI. Another issue with the use of BALB/c mice as a model organism is the different pathology of the disease compared to human, where the formation of granulomas (multicellular structures resulting from the immune response) is typical for the most common pulmonary form of TB. Pulmonary granulomas impose a significant barrier to drug permeation, with the diffused highly protein-rich lipophilic molecules. The use of C3HeB/FeJ mice, which develop granulomas during infection, appears to be a more advantageous model organism that provides more accurate information, yet most currently developed compounds continue to be tested using BALB/c mice model^178^, ^179^, ^180^, ^181^, ^182^. To promote the design of novel anti-TB agents, considering the complex environment in which the compounds must permeate before reaching the target cellular structure, a new metric has been proposed. This approach addresses the prediction of compound binding to caseum, which is pivotal for estimating permeation through the caseous environment of granulomas and necrotic lesions. The caseum binding is predicted based on *c*log*P*, the count of aromatic rings, and the count of *sp*^2^ carbons in the molecule^181^. Despite this obstacle and slow progress, the pharmaceutical landscape has witnessed a surge in enthusiasm for the development of innovative anti-TB agents. Currently, 19 promising drug candidates are undergoing clinical trials. Still, there is a scarcity of promising agents boasting novel MOA against MDR-TB infections.

Phenotypic drug discovery is one of the main strategies used in the development of new anti-TB agents, yet it is hampered by difficulties in elucidating the specific MOA. A technique used to resolve this issue is the generation of spontaneous drug-resistant mutants and their subsequent genome sequencing using data analysis. This approach is not always successful; therefore, for some candidates, their MOA remains elusive^183^. Other compounds are difficult to categorize due to the complexity of their MOA, aiming at multiple targets simultaneously. Although nearly all small molecules undergoing concurrent clinical evaluation have been identified using this method, additional obstacles persist beyond the identification mentioned above. Firstly, a significant portion of these identified hits acts on known targets for which cases of resistant infections are documented, thereby posing a high risk of cross-resistance, minimizing the attractiveness of such hits^184^. Another challenge is the difficulty in translating *in vitro* activity into *in vivo*^185^; a large proportion of identified new anti-TB agents fail at this step, partly due to the routine applied for cultivation in a nutrient-rich broth, which does not mimic the real environment within a living organism^186^. Many scientists have long endeavored to address these challenges by developing novel screening methods. One promising approach involves assessing the efficacy in *Mtb*-infected macrophages. While considerably more costly and complex, the data obtained provide significantly better insights into potential *in vivo* efficacy, primarily due to assay conditions closely resembling real-life scenarios within a living organism. Additionally, this approach offers insight into the toxicity of tested compounds towards mammalian cells^187^^,^^188^. The discovery of telacebec is a successful example of *Mtb*-infected macrophage testing applied for this drug^137^^,^^138^.

Another successful approach in the development of new anti-TB drugs is the repurposing of known therapeutics, wherein minor structural modification generates a new lead candidate capable of exhibiting efficacy against *Mtb* while maintaining the safety profile of the parent compound^189^. Sanfetrinem, an orally available tricyclic *β*-lactam antibiotic, is an example of the anti-TB clinical candidate discovered by this approach^190^. MBX-4888A was invented by delivering subtle structural modifications to spectinomycin. Indeed, MBX-4888A revealed decreased affinity to the mycobacterial efflux pump, which resulted in a higher intracellular concentration of the drug^109^^,^^112^. Another drug explored in the same way is lansoprazole, a proton pump inhibitor, which serves as a prodrug. Reduction of the sulfoxide moiety of lansoprazole to the sulphide forms an active agent^191^.

A critical aspect of TB drug discovery is evaluating the safety profile of the compounds, particularly related to potential drug–drug interactions. This concern is associated mainly with antiretrovirals, which are essential agents to treat HIV co-infection. Ensuring the safety and efficacy of new TB drugs, especially in combination with existing therapies, is indispensable for successful treatment outcomes and the prevention of further drug resistance.

From a small molecule design perspective, 19 agents are currently undergoing clinical trials, with 8 introducing entirely new MOA. Notably, oxazolidinones dominate the clinical trials landscape, with delpazolid, sutezolid, and tedizolid in phase 2, TBI-223 in phase 1, and OTB-658 in preclinical studies. TBI-233 appears promising from a safety perspective, with a 10-fold decrease in inhibition of eukaryotic MP*S* while maintaining the same activity as LNZ. Another group of clinical candidates are BDQ derivatives, whose development was aimed primarily at reducing the risk of QT interval prolongation, and this has been achieved by TBAJ-876. TBAJ-876, currently a phase 1 drug candidate, exhibits a superior safety profile and anti-TB activity comparable to BDQ. Among drug candidates with novel MOA, DprE1 inhibitors are prominent, represented by benzothiazinone derivatives like macozinone in phase 1 and BTZ-043 in phase 2 trials. While benzothiazinones act as covalent inhibitors of DprE1, two non-covalent inhibitors, namely OPC-167832 and TBA-7371, are currently ongoing phase 2 clinical trial testing. All DprE1 inhibitors except TBA-7371 show excellent *in vitro* activity in the low nanomolar range. Furthermore, QcrB subunit inhibitors of the cytochrome *bcc* complex, such as telacebec (phase 2), and TB47 (in preclinical studies), demonstrate efficacy comparable to DprE1 inhibitors.

The number of potential new drugs in preclinical studies continues to grow, increasing the odds of identifying more compounds that could advance to clinical trials. Finally, partnerships between academia and pharmaceutical companies are vital in the development of new anti-TB drug candidates. Organizations like TB-Alliance and initiatives like GSK's Tres Cantos Open Lab Foundation are crucial in advancing TB drug discovery. TB-Alliance is a non-profit organization established in 2000 that has made a significant contribution to bridging the private, academic, and philanthropic sectors. The GSK's project enables the use of private facilities, resources, and expertise to support independent research on infectious disease medicines. Despite considerable challenges, significant advances have been made in the development of new anti-TB candidates over the past decade, providing a positive outlook for the future of TB treatment.

## Author contributions

Martin Kufa: Writing – review & editing, Writing – original draft, Visualization. Vladimir Finger: Writing – review & editing, Writing – original draft. Ondrej Kovar: Writing – review & editing, Writing – original draft. Ondrej Soukup: Writing – review & editing, Writing – original draft, Supervision, Funding acquisition. Carilyn Torruellas: Writing – review & editing, Writing – original draft. Jaroslav Roh: Writing – review & editing, Writing – original draft, Supervision, Funding acquisition. Jan Korabecny: Writing – review & editing, Writing – original draft, Visualization, Supervision, Funding acquisition.

## Conflicts of interest

Authors declare no competing interest.
